# Steroid hormones content and proteomic analysis of canine follicular fluid during the preovulatory period

**DOI:** 10.1186/1477-7827-8-132

**Published:** 2010-11-01

**Authors:** Somayyeh Fahiminiya, Karine Reynaud, Valérie Labas, Séverine Batard, Sylvie Chastant-Maillard, Nadine Gérard

**Affiliations:** 1 INRA, UMR 6175 Physiologie de la Reproduction et des Comportements, F-37380 Nouzilly, France; 2 CNRS, UMR6175 Physiologie de la Reproduction et des Comportements, F-37380 Nouzilly, France; 3 Université François Rabelais de Tours, UMR 6175 Physiologie de la Reproduction et des Comportements, F-37041 Tours, France; 4 Haras Nationaux, UMR 6175 Physiologie de la Reproduction et des Comportements, F-37380 Nouzilly, France; 5 INRA, UMR 1198 Biologie du Développement et Reproduction, F-78350 Jouy en Josas, France; 6 ENVA, UMR 1198 Biologie du Développement et Reproduction, 7 Avenue du Général de Gaulle, F - 94700 Maisons-Alfort, France; 7 INRA, UMR 6175 Physiologie de la Reproduction et des Comportements, Plate-forme d'Analyse Intégrative des Biomarqueurs, Laboratoire de Spectrométrie de Masse, F-37380 Nouzilly, France; 8 ENVA, Unité de Reproduction, 7 Avenue du Général de Gaulle, F - 94700 Maisons-Alfort, France

## Abstract

**Background:**

Follicular fluid contains substances involved in follicle activity, cell differentiation and oocyte maturation. Studies of its components may contribute to better understanding of the mechanisms underlying follicular development and oocyte quality. The canine species is characterized by several ovarian activity features that are not extensively described such as preovulatory luteinization, oocyte ovulated at the GV stage (prophase 1) and poly-oocytic follicles. In this study, we examined the hypothesis that the preovulatory LH surge is associated with changes in steroid and protein content of canine follicular fluid prior to ovulation.

**Methods:**

Follicular fluid samples were collected from canine ovaries during the preovulatory phase, before (pre-LH; n = 16 bitches) and after (post-LH; n = 16) the LH surge. Blood was simultaneously collected. Steroids were assayed by radioimmunoassay and proteomic analyses were carried out by 2D-PAGE and mass spectrometry.

**Results:**

The concentrations of 17beta-estradiol and progesterone at the pre-LH stage were 737.2 +/- 43.5 ng/ml and 2630.1 +/- 287.2 ng/ml in follicular fluid vs. 53 +/- 4.1 pg/ml and 3.9 +/- 0.3 ng/ml in plasma, respectively. At that stage, significant positive correlations between follicular size and intra-follicular steroid concentrations were recorded. After the LH peak, the intrafollicular concentration of 17beta-estradiol decreased significantly (48.3 +/- 4.4 ng/ml; p < 0.001), whereas that of progesterone increased (11690.2 +/- 693.6 ng/ml; p < 0.001). Plasmatic concentration of 17beta-estradiol was not modified (49 +/- 9.6 pg/ml) after the LH peak, but that of progesterone significantly increased (9.8 +/- 0.63 ng/ml).

Proteomic analysis of canine follicular fluid identified 38 protein spots, corresponding to 21 proteins, some of which are known to play roles in the ovarian physiology. The comparison of 2D-PAGE patterns of follicular fluids from the pre- and post-LH stages demonstrated 3 differentially stained single spot or groups of spots. One of them was identified as complement factor B. A comparison of follicular fluid and plasma protein patterns demonstrated a group of 4 spots that were more concentrated in plasma than in follicular fluid, and a single spot specific to follicular fluid. These proteins were identified as gelsolin and clusterin, respectively.

**Conclusion:**

Our results provide the first demonstration of size-related changes in the steroid concentrations in canine follicular fluid associated with the LH surge. 2D protein mapping allowed identification of several proteins that may play a role in follicle physiology and ovarian activity at the preovulatory stage. This may help in the future to explain and to better understand the species specificities that are described in dogs.

## Background

Follicular fluid is the microenvironment in which the cumulus-oocyte complex matures and somatic cells (granulosa and theca cells) proliferate and differentiate. It was suggested that the follicular fluid originates from both somatic cells, which produce factors related to their metabolic activity [1] and from plasma which enters the extra-vascular spaces, as well as the antrum of follicles [2]. Furthermore, it contains a number of soluble factors implicated in various stages of follicular development. The study of its components may contribute, at least in part, to our understanding of the mechanisms underlying this process.

In mammalian species, the preovulatory maturation of the oocyte and follicle is under the control of gonadotropin hormones synthesized by the anterior pituitary gland. In most species, the sudden surge in luteinizing hormone (LH) induces morphological and functional changes in the ovulatory follicle that result in granulosa and theca cells differentiation in preparation of follicle rupture and corpus luteum formation, in expansion of cumulus cells, and in the resumption of meiotic maturation of the oocyte. Some coordination between these events is required to achieve the production of a mature oocyte suitable for fertilization. This should also be essential for subsequent embryonic development and formation of a corpus luteum capable of supporting early pregnancy.

In the bitch, the hormonal peaks of follicle stimulating hormone (FSH) and LH are observed before ovulation [3] and LH levels reach 9 ng/ml (2-22 ng/ml; [4]). Nevertheless, the ovarian physiology in the bitch compared to that of other mammals exhibits at least three major uncommon features. Firstly, the endogenous LH peak is very lengthy since it lasts 48-72 h [3,4], and preovulatory luteinization is observed in dogs, with an increase in plasma progesterone level prior to the LH peak [3,4]. Secondly, around 40% of the follicles in the canine ovary are poly-oocytic. Some may contain as many as 17 oocytes [5,6]. But the most striking feature of canine ovaries is that oocytes are ovulated at an immature (GV; prophase I) stage and reach the fertilizable metaphase II stage only after maturation for 56-72 h within the oviduct [7,8]. Several mechanisms may explain the later feature: the preovulatory canine oocytes, in contrast to those of other mammals, may be incompetent for resumption of meiosis by themselves [9] or the canine follicular cells may be are unable to transduce the ovulatory signal to the oocyte. It is also possible that the follicular fluid either does not contain the adequate factors for achievement of oocyte maturation or contains meiosis inhibitory substances. Nevertheless, at the present time, no information is available in the literature about the composition of the canine follicular fluid.

It should be noted that in vitro technologies such as oocyte maturation (IVM) or fertilization (IVF) and subsequent embryo development are particularly difficult to obtain in canine. Actually, the rate of success in IVM is still very low (10-20%) and a high rate of polyspermy is observed during IVF [10]. These disappointing results may be related to the immaturity of the canine oocytes collected for in vitro studies. It has been speculated that these oocytes, even if collected in vivo at the pre-LH or post-LH stages, may be incompetent for IVM and IVF [9]. Indeed, even when preovulatory (post-LH) oocytes collected in vivo are cultured in vitro, the IVM rate remains quite low (maximum 32%; [11]). A better understanding of the factors required by the canine oocytes to complete their growth and resume meiosis in vivo, as well as putative meiosis inhibitor factors, would be of help in developing an adequate IVM medium, and might thus, to overcome the main limiting factor of assisted reproduction techniques in this species.

In this study, we examined the hypothesis that the preovulatory LH surge is associated with changes in steroid and protein content of canine follicular fluid prior to ovulation. For this purpose, we first determined the levels of steroids (17beta-estradiol and progesterone) present in the canine follicular fluid in vivo, before and after the LH surge, then investigated the protein composition of the follicular fluid during the preovulatory phase, with special emphasis on the potential effect of LH. We also attempted to characterize specific differences in protein composition of follicular fluid and plasma in order to identify proteins that accumulate or are missing in canine follicular fluid. These proteins may hold the key to the reproductive process and further evaluation may be useful in the search of potential biomarkers of follicle/oocyte quality, or as factors responsible of the canine ovarian features.

## Methods

### Animals and monitoring of oestrous cycles

Thirty-two Beagle bitches (3.3 +/- 0.5 years old) from our experimental kennel were included in this study. Ovarian cycles were assessed by blood progesterone assays and by vaginal smears coupled with Harris-Shorr staining and eosinophilic indexes determination [12]. For progesterone assays, the blood samples were collected at the cephalic or jugular vein on lithium heparinate, centrifuged and plasma was stored at -20°c until use. Plasma progesterone was assayed by enhanced chemi-luminescence Elecsys kit (Roche Diagnostics, Meylan, France). Intra-assay and inter-assay coefficients of variation were less than 2% [8].

Progesterone assays were carried out every other day as long as the percentage of superficial cells remained below 80% and daily once that percentage exceeded 80% ("oestrus" vaginal smears).

As soon as the progesterone level reached 0.5 ng/ml, blood was collected 3 times a day and plasma was stored at -20°C until assayed for LH concentration. Once the progesterone concentration exceeded 2 ng/ml, ovarian transabdominal ultrasonography (ultrasonograph HDI 3500, probe 7.5 MHz; ATL, Philips Systèmes Médicaux, Suresnes, France, 0.19 mm in resolution) was carried out two to three times a day to assess follicular growth, as previously described [8].

Bitches were assigned a posteriori to one of two experimental groups: in the pre-LH group (n = 16 bitches), bitches had an oestrus vaginal smear, no LH rise was detectable in plasma samples and follicles larger than 2 mm were visible by ultrasonography. In the post-LH group (n = 16 bitches), bitches displayed an oestrus vaginal smear, LH peak had occurred and antral follicles were still visible at ultrasonography, demonstrating that ovulation had not started yet.

This protocol was approved by the Ethics Committee of the "Alfort National Veterinary School" (France).

### Collection of follicular fluids and plasmas

Ovariectomies were performed during the preovulatory phase using a conventional surgical procedure [13]. The ovaries were collected from excised ovarian bursa. On gross morphological evaluation, visible follicles were counted and measured [14]. All follicles larger than 2.5 mm were punctured individually with stretched Pasteur pipettes. The collected follicular fluids were observed under a stereomicroscope to recover the cumulus-oocyte-complexes. Evaluation of the cumulus mucification was used in parallel to the LH assay, to confirm the classification of canine follicles in pre-LH (no mucification) or post-LH (mucification occurred) stage. Only follicular fluids free from blood contamination were kept and were stored after centrifugation (10 min at 5000 g) to eliminate cells and other contaminants. Fluid supernatants were immediately frozen at -20°C. Peripheral blood was also collected at the time of ovariectomy, centrifuged, and plasma samples were stored under the same conditions as follicular fluids, until further analysis.

### Steroid hormones assays in canine follicular fluids and plasmas

Follicular fluids were individually assayed for progesterone and 17beta-estradiol by radioimmunoassay (RIA), without prior extraction. Progesterone assay was performed as previously described [15,16]. 17beta-estradiol was assayed with the kit [125I] E2 DIASORIN (Stillwater, Minnesota, USA). For assays in plasma, the steroids were extracted before either assay.

### Protein assay and 2D-PAGE analysis of canine follicular fluid and plasma

In order to study the effect of the LH surge on the protein profile of canine follicular fluid, follicular fluids were collected from follicles larger than 4.5 mm at pre-LH stage (n = 4 from 3 bitches) and at post-LH stage (n = 4 from 4 bitches) and were analyzed by 2D-PAGE. To compare the 2D-PAGE protein patterns of follicular fluid and plasma, samples of 4 bitches from pre-LH group were considered; follicular fluids (n = 4) being collected from follicles between 4.5 and 5.5 mm. All follicular fluid samples used for 2D-PAGE were free from blood contamination. All the chemicals and materials used for proteomic studies were purchased from Bio-Rad (Marne-la-Coquette, France), unless otherwise indicated. The total protein content of crude canine follicular fluid and plasma samples was determined using a commercial protein assay kit (DC Protein Assay) with bovine serum albumin as standard (Pierce, Rockford, Illinois, USA) according to manufacturer's instructions. 2D-PAGE analysis of canine follicular fluids was performed as previously described by Fahiminiya et al. [17]. Briefly, 100 micrograms of proteins were diluted in a hydration solution that was absorbed actively into ReadyStrip™ IPG (11 cm Immobilized pH Gradient IPG strips, pH range 3-10). After protein separation by isoelectric focusing (IEF), strips were equilibrated for 15 min with SDS-PAGE equilibration buffer, in order to resolubilize the proteins and to reduce disulfide bonds. In a second step, the same equilibration buffer containing 2.5% w/v iodoacetamide was used for 15 min to block free sulfhydryl groups. IPG strips were then subjected to SDS-PAGE (10%). The 2D gels were stained with a mass spectrometry compatible silver nitrate staining [18].

### 2D-PAGE image analysis

Stained gels were scanned and digitalized at 16-bit resolution using ImageScanner (Amersham Pharmacia Biotech, GE Healthcare Europe GmBH, Orsay, France). The resulting TIFF images were analyzed with Progenesis software (version 2008; Nonlinear Dynamics Ltd, Newcastle upon Tyne, UK), as previously described by Fahiminiya et al. [17].

### Identification of proteins from 2D-PAGE by mass spectrometry

Stained protein spots were excised from the gels, cut into small blocks and digested with trypsin, as previously described by Shevchenko et al. [18]. The digested peptide fragments were sequenced by nano-LC-MS/MS (nanoscale capillary liquid chromatography-tandem mass spectrometry) using ion trap mass spectrometers (GE Healthcare, Europe GmBH, Munich, Germany). The Ettan MDLC controlled by UNICORNTM software (GE Healthcare, Europe GmBH, Munich, Germany) was used for desalting and separation of tryptic peptides prior to online MS and MS/MS ion trap analyses. Each sample was desalted using a pre-column (Zorbax 300-SB C18 trap column, 300 μm i.d × 5 mm, Agilent Technologies, Massy, France). Peptides were separated on a C18 column (Zorbax 300-SB C18, 75 μm i.d × 150 mm, Agilent Technologies) using a flow rate of 300 nL/min and applying a gradient of buffer A (0.1% formic acid) and buffer B (0.1% formic acid/84% acetonitrile). Eluted peptides were analyzed online with LTQ Linear Ion Trap Mass Spectrometer (Thermo Electron, San Jose, CA, USA). Each scan cycle consisted of one full scan mass spectrum (m/z 400-2000) collected in centroïd mode followed by three MS/MS events in centroïd mode. For CID spectra (MS2), the isolation width was 2 m/z units and the normalized collision energy was 40%. Dynamic exclusion was activated during 30 s with a repeat count of 1.

The acquired MS/MS spectra were automatically searched against the non-redundant Uniprot SwissProt database using the TurboSEQUEST (Thermo Finnigan, San Jose, CA, USA) program in the BioWorksTM 3.1 software suite (Thermo Finnigan, San Jose, CA, USA). The databank search parameters allowed 2 tryptic missed cleavage and included oxidized methionine (+16 Da) and carbamidomethylation on cystein (+57 Da) as variable modifications. The output data were evaluated in term of Xcorr magnitude up to 1.7, 2.2 and 3.5 for charge states 1+, 2+ and 3+ respectively and a minimum of two peptides. In the case of identification with only one peptide, MS/MS spectrum was visually verified.

### Statistics

For steroid analysis, results are expressed as mean +/- SEM. Results were considered significant when P < 0.05. Effects of the ovarian stage (pre-LH and post-LH) on steroid content were analyzed by a t-test. Effects of follicle size on steroid contents and relationship between plasma and follicular fluid steroids were analyzed with a linear regression and Pearson's correlation coefficient (software SAS, Cary, NC, USA). For proteomic analysis, means of spot volumes in each group of samples were compared after normalization, using the ANOVA statistical package included in SameSpots. Only differences with P < 0.01 for random occurrence were considered to be significant, and only spots that were statistically different were selected for analysis.

## Results

### Follicle features and steroids content in canine follicular fluid and plasma

The diameter of follicles ranged between 2.5 mm (limit of collection) and 6.5 mm in the pre-LH group (72 follicles), and between 2.5 mm and 7 mm in the post-LH group (73 follicles). Out of them, 9 follicles were found poly-oocytic (7 at the pre-LH stage and 2 at the post-LH stage, all between 4.5 and 6.5 mm in diameter). The volume of follicular fluid collected was between 2 and 45 μl at the pre-LH stage and between 2 and 70 μl at the post-LH stage. Follicular fluid volume was correlated with the follicle diameter (r = 0.63 p < 0.01 and r = 0.54 p < 0.01 at the pre-LH and the post-LH stage, respectively).

#### 17beta-estradiol

**Figure 1:**
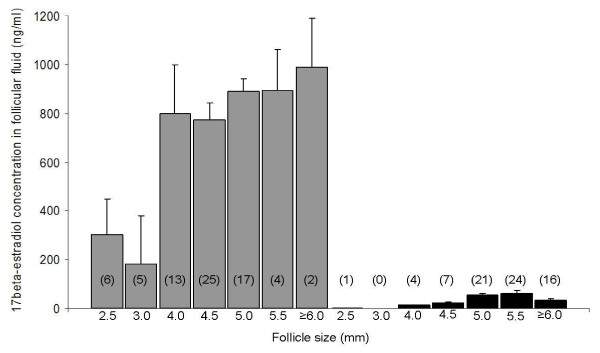
**17beta-estradiol concentration in the fluid of canine follicles**. Canine follicular fluid was collected at the pre-LH stage (grey, n = 72) and at the post-LH stage (black, n = 73). Number of follicular fluids assayed is indicated within round brackets

17beta-estradiol concentration was determined in 72 follicular fluid samples collected at the pre-LH stage and in 73 follicular fluids collected at the post-LH stage (Figure 1).

At the pre-LH stage, the mean intrafollicular concentration of 17beta-estradiol was 737.2 +/- 43.5 ng/ml (ranging from 21.7 to 1590.4 ng/ml). A significant positive correlation was observed between follicle size and intrafollicular 17beta-estradiol concentration, (r = 0.53; p < 0.0001). Indeed, the 17beta-estradiol concentration was significantly lower (p < 0.001) in follicles < 4.0 mm (n = 11; 247.0 +/- 89.9 ng/ml) than in follicles ≥ 4 mm (n = 61; 832.5 +/- 44.0 ng/ml). Furthermore, no significant difference was observed between 17beta-estradiol concentrations measured in follicular fluids collected from mono-oocytic vs. poly-oocytic follicles at this stage (725.1 +/- 42.0 ng/ml vs. 828.6 +/- 147.2 ng/ml, respectively). The levels of estradiol observed in polyovular follicles (ranging from 159.8 to 1295.3 ng/ml) were not different from those of mono-ovular follicles.

At the post-LH stage, the mean intrafollicular concentration of 17beta-estradiol was 48.3 +/- 4.4 ng/ml (ranging from 4.1 to 161.5 ng/ml). No significant correlation between follicle size and 17beta-estradiol concentration was observed (p = 0.96). The decrease in intrafollicular 17beta-estradiol concentration after the LH surge was highly significant (p < 0.001). In poly-oocytic follicles, the level of 17beta-estradiol was in the same range of concentration (14.6 and 27 ng/ml).

**Figure 2:**
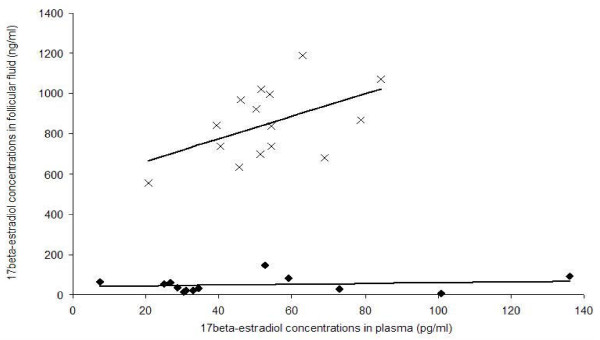
**Correlation between 17beta-estradiol concentration in plasma and in follicular fluid**. Correlations were calculated at the pre-LH stage (crosses) and at the post-LH stage (black rhombs)

In order to better characterize the steroid content in dogs, plasma levels of 17beta-estradiol at the time of ovariectomy were compared to intrafollicular levels. The mean plasmatic 17beta-estradiol concentration was 53.5 +/- 4.1 pg/ml at the pre-LH stage and 49.0 +/- 9.6 pg/ml at the post-LH stage (p > 0.05). Intrafollicular 17beta-estradiol levels (ng/ml) were between 9884 and 26969 times higher than plasma levels (pg/ml) at the pre-LH stage and from 84 to 8670 times higher than plasma levels at the post-LH stage. As shown in Figure 2, intrafollicular and plasmatic 17beta-estradiol concentrations tended to be correlated at the pre-LH stage (p = 0.06).

#### Progesterone

**Figure 3:**
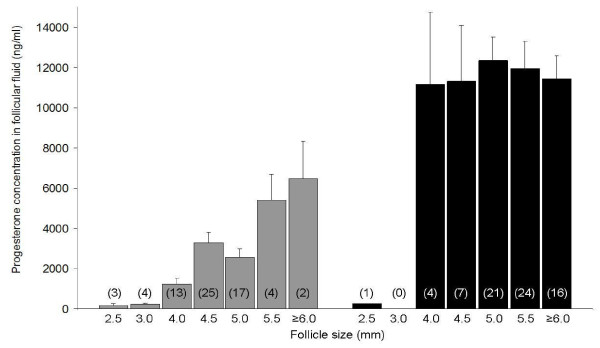
**Progesterone concentration in the fluid of canine follicles**. Canine follicular fluid was collected at the pre-LH stage (grey, n = 68) and at the post-LH stage (black, n = 73)

Progesterone was assayed in 68 follicular fluid samples collected at the pre-LH stage and in 73 follicular fluid samples collected at the post-LH stage (Figure 3). Actually, progesterone was not assayed in 4 follicles < 4 mm at the pre-LH stage because the volume of follicular fluid collected was too small. Those non-assayed follicles were 2.5 mm (n = 3) and 3 mm (n = 1) sized and originated from 4 different bitches.

At the pre-LH stage, the mean intrafollicular concentration of progesterone was 2630.1 +/- 287.2 ng/ml (ranging from 34.3 to 9205.3 ng/ml). A significant positive correlation was found between follicle size and intrafollicular progesterone concentrations (r = 0.15; p < 0.0001). In follicles larger than 4 mm (n = 61), the mean progesterone concentration was 2918.7 +/- 299.8 ng/ml. Progesterone concentrations were not significantly different between mono-oocytic and poly-oocytic follicles (2590.7 +/- 305.0 ng/ml vs. 2962.3 +/- 910.5 ng/ml, respectively).

At the post-LH stage, the intrafollicular concentration of progesterone was 11690.2 +/- 693.6 ng/ml (ranging from 245.4 to 28096.5 ng/ml) and no significant correlation between follicle size and progesterone concentration was detected (p = 0.22). In the single follicle with a diameter below 4 mm, the progesterone concentration in the fluid was dramatically lower (245 ng/ml) than that detected in follicles ≥ 4 mm (11849.5 +/- 684.5 ng/ml). The increase in intrafollicular progesterone concentration after the LH surge was highly significant (p < 0.001).

**Figure 4:**
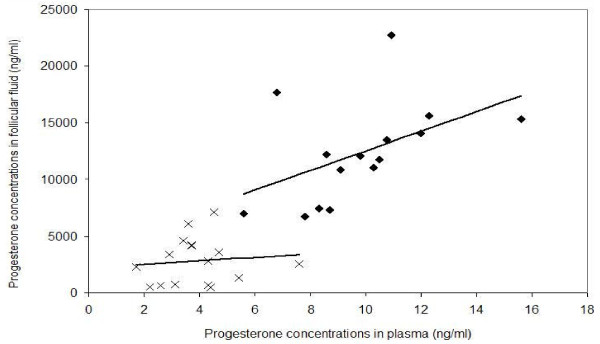
**Correlation between progesterone concentration in plasma and in follicular fluid**. Correlations were calculated at the pre-LH stage (crosses) and at the post-LH stage (black rhombs)

The plasmatic levels of progesterone were significantly higher at the post-LH stage (9.80 +/- 0.63 ng/ml) than at the pre-LH stage (3.9 +/- 0.3 ng/ml; p < 0.05). Intrafollicular progesterone levels were between 100 and 1693 times higher than in plasma at the pre-LH stage and from 843 to 2606 times higher than in plasma at the post-LH stage. As shown in Figure 4, intrafollicular and plasmatic progesterone concentrations tended to be correlated at the post-LH stage (p = 0.07, Figure 4).

### Proteomic analysis of canine follicular fluid and plasma

The 2D-PAGE methodology was used to characterize the protein profile of canine follicular fluid and to compare the protein patterns of: A) follicular fluids at pre-LH (size of follicles: 4.75 +/- 0.25 mm) vs. post-LH (size of follicles: 5.19 +/- 0.12 mm; p > 0.05) stages and B) follicular fluid vs. plasma. For this purpose, follicular fluids collected from follicles larger than 4.5 mm at pre-LH stage (n = 4) and at post-LH stage (n = 4), all from 7 different bitches were used. To compare the 2D-PAGE protein patterns of follicular fluid and plasma, samples of 4 bitches from pre-LH group were considered; follicular fluids (n = 4) being collected from follicles between 4.5 and 5.5 mm. All follicular fluid samples used for 2D-PAGE were free from blood contamination.

#### 2D proteins profile of canine follicular fluid

**Figure 5:**
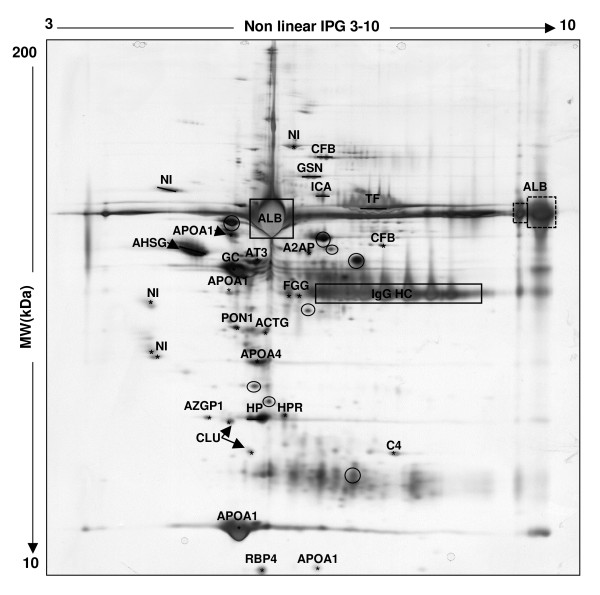
**Silver stained 2D-PAGE profile of crude canine follicular fluid**. 100 μg of proteins samples were applied to a non-linear pI 3-10 IPG strip in the first dimension and separated on a 10% SDS-PAGE gel in the second dimension (Molecular weight 200-10 kDa range). The positions of the high-abundant proteins albumin (ALB) and immunoglobulin heavy chain (HV01 and HV02), are indicated by dark full and dotted rectangles. Additional albumin fragments are indicated by dark circles (see Additional file 1, Table S1). All other identified proteins are named on the figure and their characteristics are shown in Table 1. NI: non-identified proteins

Slightly more than 1000 protein spots with molecular weight (Mr) between 10 and 200 kDa were detected by silver nitrate staining after IEF/PAGE (3.0 < isoelectric point pI < 10) separation (Figure 5). Several protein spots were excised from the gel and analyzed by mass spectrometer for identification. Among them, 38 spots were identified, corresponding to 21 different proteins. The high-abundant proteins albumin (ALB) and immunoglobulin heavy chains (HV01 and HV02), appear as intense and large spots in canine follicular fluid protein patterns (indicated by dark rectangles in Figure 5). Albumin was identified in 10 of the collected spots, mainly as fragments (indicated by dark circles in Figure 5: 67 < Mr < 28; 5.07 < pI < 6.82; see Additional file 1, Table S1). Two other spots also identified as albumin most probably correspond to the native protein that has not correctly entered into the IEF gel (indicated by dotted rectangle in Figure 5). All other protein spots that have been identified in canine follicular fluid are shown in Figure 5 and their characteristics (Uniprot accession code (http://www.ebi.uniprot.org/index.shtml), Mr and pI are indicated in Table 1. Among them, 4 spots were identified as apolipoprotein A-1, and 2 spots as clusterin, complement factor B, inhibitor of carbonic anhydrase and haptoglobin. Most of the identified proteins are known to be secreted proteins, whereas 2 other are mostly known as cytosolic, such as actin and gelsolin, which are described as cytoskeleton proteins.

**Table 1:** Identification by mass spectrometry of canine follicular fluid proteins separated by 2D-PAGE

Gene	**AC**^**1 **^**N°**	Protein name	**Exp**^**2**^	**Theo**^**2**^	**Score**^**3**^	**P N°**^**2**^	**SC (%)**^**4**^
			**pI**^**2 **^**/Mr**^**2**^				
A2AP	P28800	Alpha-2-antiplasmin	6,08/57	5.36/54	10.17	1	2.2
ACTG	Q9UVW9	Actin-gamma	5,49/42	5.36/41	20.22	2	4.8
AHSG	P12763	Alpha-2-HS-glycoprotein	4,66/57	5.16/38	10.14	1	3.6
APOA1	P02648	Apolipoprotein A-I	5,05/48	5.06/30	10.16	1	3.4
APOA1	P02648	Apolipoprotein A-I	5,07/24	5.06/30	296.41	30	72.2
APOA1	P02648	Apolipoprotein A-I	6,20/21	5.06/30	50.23	5	24.1
APOA1	P02648	Apolipoprotein A-I	5,07/62	5.06/30	40.24	4	14.3
APOA4	Q32PJ2	Apolipoprotein A-IV	5,44/38	5.17/42	50.21	5	9.7
AT3	P41361	Antithrombin-III	5,14/55	7.08/52	80.20	8	8
AZGP1	Q3ZCH5	Zinc-alpha-2-glycoprotein	4,8/33	4.98/33	30.14	3	10.4
C4	P01030	Complement C4	7,48/31	6.13/101	34.19	4	4
CFB	P81187	Complement factor B	7,04/59	7.07/85	20.21	2	3.8
CFB	P81187	Complement factor B	6,38/102	7.5/85	20.26	2	3.8
CLU	P25473	Clusterin	5,05/33	5.56/51	10.16	1	3.6
CLU	P25473	Clusterin	5,3/31	5.56/51	20.16	2	6.1
FGG	P02679	Fibrinogen gamma chain	5,8/48	5.27/51	56.25	6	14.3
GC	Q3MHN5	Vitamin D-binding protein	5,17/54	5.24/53	70.29	7	9.5
GSN	Q3SX14	Gelsolin	6,01/88	5.45/80	20.24	2	5.5
HP	P19006	Haptoglobin	5,31/33	5.68/36	60.21	6	15.8
HP	P19006	Haptoglobin	5,47/33	5.68/36	50.30	5	21
HPR	Q28801	Haptoglobin-related protein	5,75/33	7.61/38	10.15	1	5.8
ICA	Q29545	Inhibitor of carbonic anhydrase	6,29/77	5.85/77	50.20	5	3.6
PON1	P27169	Paraoxonase/arylesterase 1	5,13/42	4.96/39	20.27	2	7.9
RBP4	P18902	Retinol-binding protein 4	5,45/20	5.33/21	40.21	4	12
TF	Q29443	Serotransferrin	7,05/72	6.69/77	42.25	5	7.7

The experimental position of identified spots is shown in Figure 5.

^1^ AC is an accession number from the UniProt database

^2^ Exp: Experimental; Theo: Theoretical; pI: Isoelectric point; Mr: Molecular weight; P N°: Peptid number; SC: Sequence coverage

^3^ Score is −10*Log (P), where P is the probability that the observed match is a random event

^4^ Sequence coverage (%) is based on number of peptide masses matched in UniProt databases

#### Potential effect of the LH surge on the protein profile of canine follicular fluid

**Figure 6:**
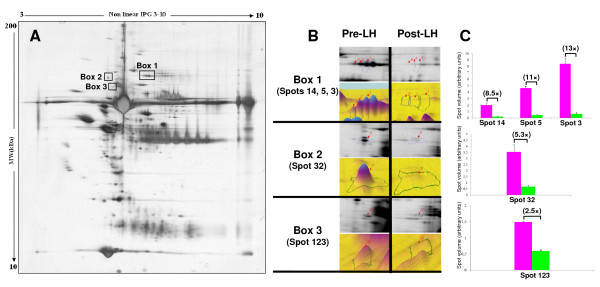
**Computerized 2D-PAGE pattern analysis and comparison of pre-LH and post-LH canine follicular fluid**. (A) 2D-PAGE profile of follicular fluid at the pre-LH stage and position of differential protein spots with the post-LH stage (dark rectangles). (B) Magnified and 3D images of differentially expressed protein spots of boxes 1, 2 and 3 (indicated by dark rectangles in A). (C) Bar charts indicates the relative spot volume (Mean +/- SEM) and fold change between both stages. Spots of box 1 have been identified as complement factor B

Computerized 2D protein pattern analysis was performed in order to compare 2D profiles at the two stages (pre- and post-LH) and to detect the differentially expressed proteins. We observed a high similarity in the 2D protein patterns before and after the LH peak, with only one group of spots and two singles that displayed significantly different levels of staining (indicated by rectangles in Figure 6A). The magnified images of the differential spots are presented in Figure 6B, and the statistical analysis of the quantified spot volumes (% volume) is shown in Figure 6C. The volumes of these spots are lower at the post-LH stage, by a magnitude of 2.5 (spot 123; Figure 6C) to 13 (spot 3; Figure 6C). Protein spots numbered 3, 5 and 14 corresponding to box 1 (Figure 6A) were identified as complement factor B, which has previously been detected in human follicular fluid [19].

#### Comparison between canine follicular fluid and plasma protein patterns

**Figure 7:**
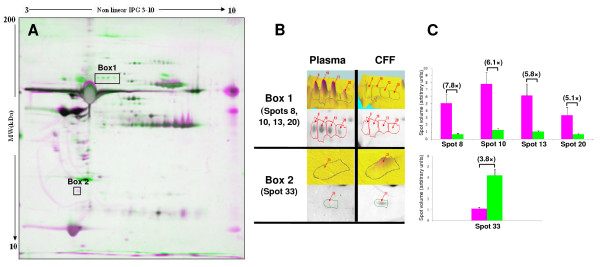
**Computerized 2D-PAGE pattern analysis and comparison of canine follicular fluid and corresponding plasma samples**. (A) Matching image of the 2D-PAGE profiles of plasma (in purple) and follicular fluid (in green). The positions of differentially expressed spots are indicated by dark rectangles. (B) Magnified and 3D images of differentially expressed protein spots of boxes 1 and 2 (indicated by dark rectangles in A). (C) Bar charts indicates the relative spot volume (Mean +/- SEM) and fold change between both biological fluids. Spots of box 1 and 2 have been identified as gelsolin and clusterin, respectively

Follicular fluid and plasma samples from the same bitches (n = 4; pre-LH stage) were analyzed by 2D-PAGE. Computerized comparison of 2D patterns allowed detection of differentially stained protein spots between these two biological fluids (Figure 7A). One group of spots (box 1) and a single spot (box 2) were found differentially expressed between plasma and follicular fluid, as demonstrated by the magnified images and quantified spot volumes presented in Figures 7B and 7C, respectively. Spots from box 1 (spots n° 8, 10, 13 and 20) were identified as Gelsolin. They were found at higher levels in plasma (7.8, 6.1, 5.8 and 5.1 fold increases, respectively) than in follicular fluid. Spot number 33 (box 2) was identified as a small isoform of clusterin which is in higher amount in follicular fluid than in plasma (3.8 fold increase).

## Discussion

During follicular growth and maturation, the follicular fluid is the microenvironment of follicular cells and of oocyte. It contains substances presumably implicated in cell differentiation, rupture of the follicular wall, and gamete quality. One can reasonably expect that the determination of its steroid content and protein composition will contribute to a better understanding of ovarian physiology and of regulation of follicular growth and maturation. The canine species is characterized by several ovarian activity features that are not extensively described and well known yet, such as preovulatory luteinization; oocyte ovulated at GV (prophase 1) stage and poly-oocytic follicles. The present study was designed to characterize the steroid and, for the first time, the proteomic content of canine follicular fluid and plasma. These may help in the future to explain and to better understand the species specificities that are described in dogs. In our investigation, 2D-PAGE study of canine follicular fluid revealed few differentially expressed proteins between follicular fluid and plasma (gelsolin, clusterin), and between follicular fluid collected before or after the endogenous LH surge (complement factor B and some unidentified proteins).

As previously mentioned, one of the main peculiarities of the canine ovarian physiology is the preovulatory luteinization [3], which is clearly observed in our study, with a significant increase in plasmatic progesterone (nearly 2.5 fold) between the pre- and the post-LH stages. The plasmatic levels of progesterone, but also of 17beta-estradiol, found in our study are similar to the ones previously reported by others [20,21] and by our own group [4]. However, the most important question is not the plasmatic, but the intrafollicular levels of progesterone/17beta-estradiol, i.e. the level to which the oocytes are directly exposed. Almost no published data are available regarding the steroid content of canine follicular fluid. To our knowledge, a single study has been carried out earlier (Metcalfe, unpublished data [22]) in Labrador bitches at the post-LH stage, with no precise description of punctured follicle sizes (from 5 to 11 mm in diameter). Our study is the first describing of intrafollicular levels of 17beta-estradiol and progesterone in parallel to blood concentrations with precise characterization of follicular diameter and stage (pre- and post-LH stages) in the canine species.

As recorded previously in several mammalian species, concentrations of 17beta-estradiol and progesterone were found to be dramatically lower in blood than in follicular fluid. This may be due to the fact that in dogs like other species, the follicle is a major site of steroid synthesis, and that steroids are mostly secreted and concentrated in follicular fluid, before entering blood flow. Nevertheless, we found no data regarding the ovarian localisation and regulation of steroid synthesis and secretion in the canine species. We observed that the concentration of 17beta-estradiol in follicular fluid dropped significantly after the LH peak whereas that of progesterone increased significantly, which is consistent with findings in other mammals (sheep: [23]; pig: [24]; cow: [25]; human: [26]; horse: [27]). We can assume that this reflects either a lower/higher level of the enzymes involved in steroid synthesis in the follicle, or to an inhibition/activation of their activity, or may be linked to substrate availability. For example the decrease in intrafollicular 17beta-estradiol after LH may be due to a decrease in aromatase level or activity, or to a decrease in androgen availability. Additionally, the increase in intrafollicular 17beta-estradiol and progesterone concentrations coincident with the increase in follicle size that we observed before the LH surge, may also be the result from steroidogenic enzyme regulation within each follicle size category. Progesterone levels recorded in the present study in canine follicular fluid are much higher than those found in sheep [28], pig [24], cow [25] and human [26]. This is probably linked to preovulatory luteinization observed in the female dog [3]. This result suggests that, in view of mimicking in vivo conditions prior to in vitro maturation of canine oocytes, one should use high level of progesterone in the culture medium [29].

Finally, the high rate of poly-oocytic follicles is a peculiarity of the ovarian physiology in dogs [5,6,30]. Recently, polyovular/poly-oocytic follicles have been reported in porcine to have a higher intrafollicular concentration of 17beta-estradiol and a lower concentration of progesterone than mono-oocytic follicles [24]. In our study, no relationship could be demonstrated between the presence of several oocytes within a follicle and 17beta-estradiol or progesterone intrafollicular levels, but the number of follicles examined was very limited.

The second objective of our study was to describe the protein composition of the canine follicular fluid during the preovulatory phase, with special emphasis on the potential effects of LH. First, we used 2D-PAGE to characterize the global protein profile of canine follicular fluid, and mass spectrometry to identify some of the proteins present in follicular fluid. By combining these two approaches, we identified 38 protein spots in the canine follicular fluid, corresponding to 21 different proteins. Our identification data are in agreement with earlier studies performed in human [31], bovine [32] and porcine [33] follicular fluids. Most of the identified proteins have previously been reported in plasma of various species [34,35], but in the present study, only follicular fluid free from blood contamination were used for the proteomic analysis. Indeed, the blood-follicular barrier is known to allow the free diffusion of molecules under 500 kDa [36]. Thus, the presence of such proteins in follicular fluid may be due to the vascular permeability of ovarian vessels during follicular growth and, at least for some of them, to local synthesis and secretion by follicular cells.

In this study, we were able to identify 11 of the protein spots in the canine follicular fluid as albumin or its fragments, and 2 as heavy chains of immunoglobulin. These two types of proteins (albumin and immunoglobulin) were reported earlier in human [31] and bovine [32] follicular fluids proteome studies. The existence of multiple forms of albumin with different isoelectric points could be due to chemical modifications of amino acid side chains [37]. Furthermore, this protein is known to function as carrier and transporter of proteins within blood and to bind physiologically important species such as lipid soluble hormones (steroid hormones), free fatty acids (apoprotein), calcium, ions (transferrin), and cytokines [38,39]. Intrafollicular albumin may participate in the transport of metabolites involved in follicular growth or in the transfer to the general circulation of some follicle specific products like steroids.

Another group of proteins that we identified in the canine follicular fluid is made up of acute phase proteins including fibrinogen gamma, haptoglobin, complement factors, apolipoprotein A-I, alpha-2-HS-glycoprotein, transferrin and retinol-binding protein 4, which are involved in inflammatory events [40,41]. Kim et al. [42] have recently shown that the level of fibrinogen gamma present in human follicular fluid may be a useful marker for the diagnosis of recurrent spontaneous abortion. Moreover, the impact of haptoglobin on women's fertility has been shown by Bottini et al. [43]. The transport of this protein into the antrum depends on the integrity of the blood-follicle barrier and might be associated with oocyte quality, possibly by interfering with the role of apolipoprotein A1 in cholesterol or vitamin E exchange between high-density lipoproteins and granulosa cells [44]. Jarkovska et al. [19] have recently showed the possible role of complement cascade and complement regulatory proteins in the maturation of the oocyte and in the development of its competence for successful fertilization.

Two types of apolipoproteins (APOA1 and APOA4) were identified in 5 protein spots on our 2D-PAGE of canine follicular fluid. These proteins are the major protein component of high density lipoprotein (HDL). APOA1 has been previously shown to be expressed in mouse granulosa cells and is regulated by progesterone receptor A (PGR-A) which is induced by LH [45]. Balestrieri et al. [46] demonstrated that haptoglobin in human follicular fluid inhibits the reverse transport of cholesterol to circulating blood by preventing the apolipoprotein A1 stimulation of the lecithin-cholesterol acyltransferase activity. These two proteins have already been observed in human follicular fluid [31].

Retinol and retinoids have been suggested to be essential for reproduction and to be involved in ovarian steroidogenesis, oocyte maturation and early embryonic development [47,48]. In the present study, a single isoform of retinol-binding protein was found in canine follicular fluid. This result is in accord with earlier observations by Anahory et al. [49], and in contrast with those of Angelucci et al. [31], who identified three different isoforms of retinol-binding protein in human follicular fluid. In canine follicular fluid, other isoforms of retinol-binding protein may be present but either spots have not been stained and collected, or spot analysis did not permit identification. Its presence in follicular fluid may be due to either passive filtration from serum across the blood follicular barrier into the follicular fluid, or to local synthesis [50].

In the present study, paraoxonase and transferrin were identified in canine follicular fluid. Both proteins are synthesized in the liver. Paraoxonase is bound and transported in plasma along with HDL. It functions as an antioxidant by preventing the oxidation of LDL (Low-density lipoproteins). Its presence has already been shown in human follicular fluid [31]. Transferrin is known as a circulating iron carrier protein, but is also produced in the testis and the ovary, where it acts as a growth factor, in addition to its role in iron endocytosis. The expression of transferrin was demonstrated in granulosa cells in human and mouse follicles [51]. In humans, the level of transferrin in the follicular fluid is highly correlated with the circulating level, suggesting that the contribution of local synthesis by granulosa cells during follicle maturation may be important.

There is plenty of evidence supporting the role of the LH surge in regulating numerous proteins implicated in ovulation and luteinization [52]. Although most of these proteins display a cellular localization, some have been localized in follicular fluid such as extracellular matrix glycoproteins [53,54], proteinases [55,56] and their inhibitors [57,58]. In the present investigation, we attempted to visualize and identify proteins in canine follicular fluid that may be modulated in response to the increase in circulating LH level. Computerized protein pattern analysis and comparison of pre-LH and post-LH canine follicular fluids demonstrated the presence of higher levels of complement factor B at the pre-LH stage. The reason why its expression decreases at post-LH stages in dog is not clear, and such a decrease has never been described previously. According to previous studies in other species, the activation of the complement system may cause a deficiency of free vascular endothelial growth factor (VEGF). As the LH surge can induce the expression of VEGF mRNA and/or protein in granulosa cells of various species [59-61], one may assume that the decrease in complement level at post-LH stage could help angiogenesis which is necessary for ovulation.

Two other protein spots that were demonstrated in our study with a lower expression at the post-LH stage could not be identified by mass spectrometry. This could be due to the low quantity of protein within spots, to the small number of cleavage sites for trypsin, which results in small number of peptides for identification. The presence of formaldehyde in the coloration method may also interfere with amine functions and modify the protein mass.

Because of the high permeability of the blood-follicle barrier, the follicular fluid content resembles that of plasma. In the present study, we hypothesize that comparison of 2D-PAGE proteins profiles of follicular fluid and plasma may reveal some proteins specific to the ovary. In this view, we demonstrated in the present study a higher level of clusterin in canine follicular fluid than in plasma. Our result is in accordance with that of Jarkovska et al. [19] who showed high levels of clusterin in human follicular fluid. Clusterin is a complement regulatory protein which plays an active role in inhibition of complement-mediated cell damage [62] and may also play a protective role in reproduction [63]. The high level of clusterin in follicular fluid might contribute to the inhibition of cytolytic activity of complement-mediated membrane attack.

Finally, we showed higher level of gelsolin in plasma than in canine follicular fluid. Circulating gelsolin is the secreted isoform of cytoplasmic gelsolin, which participates in the clearance of actin from general circulation [64]. This protein was identified earlier in human follicular fluid [65], and in the mouse ovary where it is predominantly found in the theca externa and in stromal cells [66]. In the ovary, Teubner et al. [66] proposed that gelsolin may play a role in contractile and morphogenetic processes that take place during follicular growth and ovulation via the modulation of the activity of the actomyosin ATPase. Nevertheless, intrafollicular gelsolin may also be related to actin clearance that takes place in follicular fluid and its lower concentration in canine follicular fluid compared to plasma could be explained by this function [64].

## Conclusions

This study provides the first description of 1/intrafollicular levels of steroids (17beta-estradiol and progesterone) at the pre-LH and the post-LH stages in the canine species, and 2/the protein composition of follicular fluid during the preovulatory phase in the bitch. Our results show that in canine as in other species, the intrafollicular steroid content is related to the follicle size, and is regulated at the time of the LH surge. In contrast, intrafollicular progesterone levels are much higher than those found in other mammals, most probably due to intense luteinization in canine. Of note is the fact that in canine, intrafollicular steroid levels are largely higher than blood levels. Moreover, a combination of 2D-PAGE, computing image analysis and mass spectrometry led to the identification of 21 different proteins in canine follicular fluid. Most of them are functionally interesting and are expected to play an essential role in female reproduction. We demonstrated the regulation of intrafollicular complement factor B at the preovulatory stage, and the specific levels of gelsolin and clusterin in plasma and in follicular fluid. These proteins may play a role in follicle physiology and ovarian activity at the preovulatory stage, or may be involved directly or indirectly in the regulation of the meiosis resumption. Our observation may help in the future to explain and to better understand the species specificities that are described in dogs.

## Competing interests

The authors declare that they have no competing interests.

## Authors' contributions

SF has been involved in the conception of the study, performed the 2D-PAGE experiments and computerized image analysis, organized the mass spectrometry data and drafted the manuscript. KR was involved in the conception of the study, was responsible of the collection and preparation of the samples, and supervised the steroid assays. VL performed the mass spectrometry analysis. SB participated in the collection and preparation of the samples, and performed the steroid assays. SCM was involved in the conception of the study, took care of the funding for sample collection and steroid assays, chose and performed the statistical analysis for steroid data and revised the manuscript. NG was involved in the conception of the study, took care of the funding for protein analysis and identification, supervised the proteomic analysis and performed the critical revision of the manuscript. All authors read and approved the final manuscript.

## Supplementary Material

Additional file 1 Supplemental Table 1: Characteristics of different fragments of albumin (ALB) and immunoglobulin heavy chain. Click here for file
